# The CRISPR‐Cas13a Gene‐Editing System Induces Collateral Cleavage of RNA in Glioma Cells

**DOI:** 10.1002/advs.201901299

**Published:** 2019-08-29

**Authors:** Qixue Wang, Xing Liu, Junhu Zhou, Chao Yang, Guangxiu Wang, Yanli Tan, Ye Wu, Sijing Zhang, Kaikai Yi, Chunsheng Kang

**Affiliations:** ^1^ Tianjin Medical University General Hospital Tianjin Neurological Institute Key Laboratory of Post‐neurotrauma Neuro‐repair and Regeneration in Central Nervous System Ministry of Education and Tianjin City Department of Neurosurgery Tianjin Medical University General Hospital Tianjin 300052 China; ^2^ Beijing Neurosurgical Institute Capital Medical University Beijing 100050 China; ^3^ Department of Pathology Medical College of Hebei University Baoding Hebei 071000 China

**Keywords:** collateral effect, CRISPR‐Cas13a, EGFRvIII, glioblastoma (GBM)

## Abstract

RNA is rarely used as a therapeutic target due to its flexible structure and instability. CRISPR‐Cas13a is a powerful tool for RNA knockdown, and the potential application of CRISPR‐Cas13a in cancer cells should be further studied. In this study, overexpression of LwCas13a by lentivirus in glioma cells reveals that crRNA‐EGFP induces a “collateral effect” after knocking down the target gene in EGFP‐expressing cells. EGFRvIII is a unique EGFR mutant subtype in glioma, and the CRISPR‐Cas13a system induces death in EGFRvIII‐overexpressing glioma cells. Bulk and single‐cell RNA sequencing analysis in U87‐Cas13a‐EGFRvIII cells confirm the collateral effect of the CRISPR‐Cas13a system. Furthermore, CRISPR‐Cas13a inhibits the formation of glioma intracranial tumors in mice. The results demonstrate the collateral effect of the CRISPR‐Cas13a system in cancer cells and the powerful tumor‐eliminating potential of this system.

## Graphical abstract

**It is reported that a CRISPR/Cas13a system** can exert a collateral effect after target recognition in glioma cells, indicating that the CRISPR/Cas13a system has great potential value for tumor elimination, especially for patients with specific oncogene mutations.

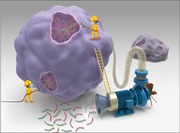

Although RNA‐based approaches have great potential, drugs targeting RNA have been hampered by the flexibility and instability of this nucleic acid.^1^ CRISPR‐Cas systems were discovered as adaptive immune systems of bacteria and archaea. Cas13a, which was initially named C2c2, is a type VI single effector protein that exhibits a RNA‐guided RNase function.^2^ After target recognition, the HEPN catalytic site is activated, and C2c2/Cas13a is triggered to degrade nonspecific RNA.[qv: 2a] Cas13 not only defends against RNA phages but also neutralizes double‐stranded DNA phages due to its trans‐RNA cleavage activities.^3^ Due to the “collateral effect” of CRISPR‐Cas13a/C2c2, it is also a promising system for nucleic acid detection.[qv: 2b,4] Applications of CRISPR‐Cas13a systems in eukaryotes are in the exploration and practice phases. In this study, we aimed to determine if the CRISPR‐Cas13a system can induce a collateral effect in human glioblastoma (GBM) cancer cells.

To improve expression efficiency, we cloned the LwaCas13a gene into a lentivirus vector and overexpressed it in U87 glioma cells. EGFP‐PEST, which is a rapidly degraded protein,^5^ was employed to test the CRISPR‐Cas13a system. We designed crRNAs against EGFP‐PEST, and we transfected these crRNAs into U87‐Cas13a‐EGFP cells and examined cellular changes (**Figure** **1**a). A time‐course immunofluorescence analysis indicated that EGFP degradation started at 4 h (Figure 1b), demonstrating the knockdown effect on EGFP by the CRISPR‐Cas13a system in U87 cells. We next measured mRNA levels in the crRNA‐transfected U87‐Cas13a‐EGFP cells, and the results were normalized to GAPDH. EGFP was significantly knocked down at 2 h, but EGFP mRNAs started to increase at 4 h (Figure 1c). We analyzed the raw data and found that the Ct value of GAPDH lost consistency starting at 4 h (Figure S1a, Supporting Information). We randomly selected HOTAIR and L3MBTL1 for quantitative reverse transcription‐polymerase chain reaction (qRT‐PCR) analysis, which revealed a significant Ct value increase of these genes (Figure S1b, Supporting Information). We speculated that this decrease in the RNA level was due to the off‐target collateral cleavage of host RNAs by Cas13a after target ssRNA activation. RNA‐denaturing gel electrophoresis of total RNA showed that the ribosomal RNA was cleaved into multiple bands starting at 4 h (Figure 1d). In addition, nontargeting crRNAs did not induce ribosomal RNA degradation in the same period (Figure 1e).

**Figure 1:**
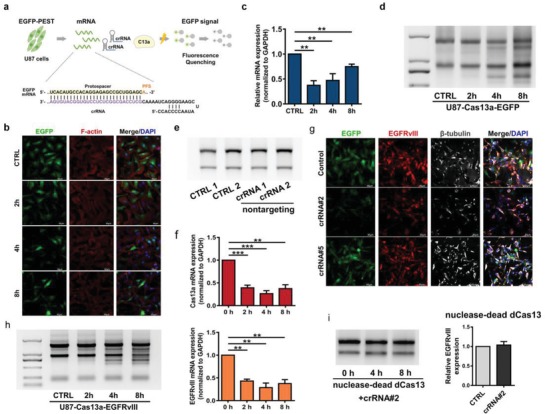
CRISPR‐Cas13a system induces comprehensive RNA interference in glioma cancer cells. a) Schematic illustration of the construction and function of CRISPR‐Cas13a in cancer cells. U87‐Cas13a‐EGFP cells were transfected with crRNA‐EGFP, and cells were collected at 0, 2, 4, and 8 h. b) Immunofluorescence (40×), c) qRT‐PCR and d) RNA‐denaturing gel electrophoresis were employed to examine changes in EGFP and total RNA. e) RNA‐denaturing gel electrophoresis was applied to examine RNA changes in U87 cells transfected with nontargeting crRNA at 8 h. f) U87‐Cas13a‐EGFRvIII cells were transfected with crRNA‐2, and qRT‐PCR analysis was applied to examine EGFRvIII and Cas13a mRNA at 0, 2, 4, and 8 h. g) U87‐Cas13a‐EGFRvIII‐EGFP cells were transfected with crRNA‐2, crRNA‐5, or Lipofectamine 3000 (control) overnight. Immunofluorescence (20×) was measured to profile the expression of EGFP, EGFRvIII, and β‐tubulin. h) Total RNA of U87‐Cas13a‐EGFRvIII cells transfected with crRNA‐2 was collected at 0, 2, 4, and 8 h, and RNA‐denaturing gel electrophoresis was employed to examine RNA integrity. i) qRT‐PCR analysis and an RNA‐denaturing gel were employed to examine EGFRvIII expression levels and the collateral effect when Cas13a was replaced by Dead‐Cas13a.

Mammalian cells have pattern‐recognition receptors for RNA viruses. The 2′,5′‐oligoadenylate (2‐5A) synthetase (OAS) can activate RNase L when viral dsRNA is recognized, which can destroy all RNA within a cell upon activation.^6^ To examine if the comprehensive RNA degradation is related to the presence of dsRNA, we transfected U87 cells with EGFP and then treated them with crRNAs. RNA‐denaturing gel electrophoresis showed the integrity of ribosomal RNA after crRNA treatment (Figure S2a, Supporting Information). Real‐time PCR analyses indicated that EGFP expression was consistent under crRNA treatment (Figure S2b, Supporting Information). We then tested the secretion of OAS1 and TNF‐α by enzyme‐linked immunosorbent assay (ELISA) and found that they were rarely impacted by crRNAs treatment (Figure S2c,d, Supporting Information).

To further examine if transduction with Cas13a lentivirus can activate the innate immune system in U87 cells, we examined OAS 1–3 and IFN‐α,β after crRNA and/or Cas13 lentiviral treatment. We used IFN‐β‐treated U87 cells as a positive control. IFN‐β treatment significantly induced the expression of OAS 1–3 in U87 cells, but crRNA, Cas13a/Dead‐Cas13a lentivirus, or crRNA combined with Cas13a lentivirus had minimal impact on OAS 1–3 protein expression (Figure S2e,f, Supporting Information). In addition, the protein expression of IFN‐α,β did not significantly change with any treatment. Thus, we concluded that the CRISPR/Cas13a system combined with crRNA does not activate the innate immune system.

GBM is the most frequent malignant brain tumor in adults.^7^ The EGFR variant III (EGFRvIII) is a persistently activated EGFR mutant subtype characterized by the junction of exon 1–8 and is unique to GBM.^8^ We designed five crRNAs to target the junction point of EGFRvIII (Figure S3, Supporting Information). qRT‐PCR indicated that crRNA‐2 (cr‐2#) had a collateral effect in U87‐Cas13a‐EGFRvIII cells (Figure 1f, and Figure S4a, Supporting Information). Real‐time cell analysis (RTCA) is a technique that measures changes in electrical impedance as cells attach and spread in a culture dish covered with a gold microelectrode array.^9^ A large spike at ≈2 h is a specific marker for U87 cells. We changed the cell media at ≈60 h, which caused a sharp decrease in the cell index. However, when the cells recovered from the medium change, the index steadily increased, except for the cr‐2# treatment group. Thus, RTCA revealed that cr‐2# transfection ultimately leads to cell death (Figure S4b, Supporting Information). Immunofluorescence analysis further confirmed that cr‐2#, but not cr‐5#, inhibited both EGFRvIII and EGFP expression in U87‐Cas13a‐EGFRvIII‐EGFP cells, resulting in dysregulated β‐tubulin morphology (Figure 1g). Furthermore, cr‐2# transfection induced ribosomal RNA cleavage in U87‐Cas13a‐EGFRvIII cells (Figure 1h). When Cas13a was replaced by a nuclease‐dead version, neither target gene knockdown nor collateral RNA cleavage was observed (Figure 1i). These results demonstrated that Cas13a is responsible for comprehensive collateral RNA cleavage by targeting EGFRvIII mRNA.

We collected total RNA from U87‐Cas13a‐EGFRvIII cells after cr‐2# transfection at 0, 2, 4, and 8 h. According to the quality control chart provided with the Agilent 2100 Bioanalyzer (Agilent, USA), the RNAs were intact in the 0 and 2 h groups. However, the integration of RNA was destroyed in a time‐dependent manner, and the ratio of 28S/18S declined to 0.8 at 8 h (Figure S5, Supporting Information). To profile the general shear pattern of CRISPR‐Cas13a, we sequenced all RNA samples of the 4 and 8 h groups despite the risk to the library building. Pearson's correlation analysis demonstrated that the sequenced samples clustered together in an intragroup manner. A low correlation was observed between the 0 and 2 h samples as well as between the 4 and 8 h samples, indicating that the collateral effect had already begun at 4 h, which is consistent with nonspecific RNase activity ex vivo[qv: 2a] (**Figure** **2**a). Principal component analysis (PCA) revealed that the samples at 8 h distributed separately from those of the other groups, suggesting that distinct transcriptomic alterations occurred after cr‐2# transfection (Figure 2b). Compared to the 0 h control, the number of differentially expressed genes increased as a function of time, while the mapping ratio to the genome decreased (Figure 2c). We clustered the RNA transcriptomes and differentially expressed genes in these groups. According to the heatmap, the expression patterns of the RNA transcriptomes started to change and several genes were differentially expressed at 4 h (Figure 2d).

**Figure 2:**
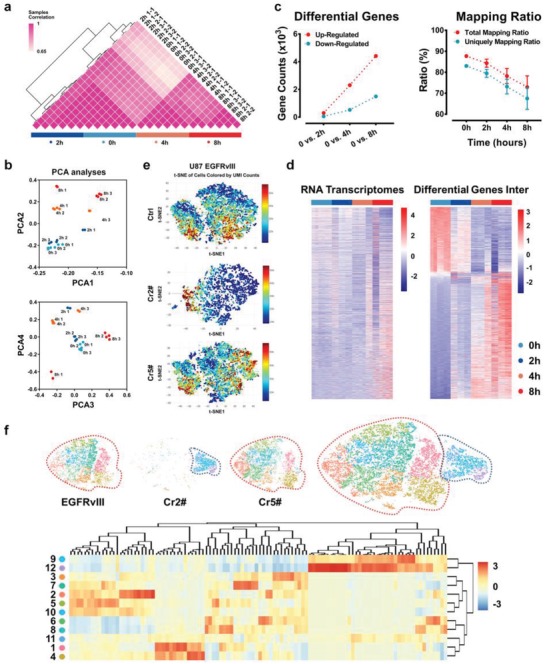
Collateral effect profiling. a) Pearson correlation, b) principal component analysis, and c) mapping ratios and differential gene expression of U87‐Cas13a‐EGFRvIII RNA‐seq samples at the indicated times. d) Heatmap of RNA transcriptomes and differential gene expression of U87‐Cas13a‐EGFRvIII RNA‐seq samples. e) T‐distribution stochastic neighbor embedding (t‐SNE) algorithm analyses of single‐cell RNA sequencing. f) Graph‐based clustering of 15507 cells reveals 12 clusters with distinct transcriptomic characteristics.

To further profile the collateral effect of the CRISPR‐Cas13a system in GBM cells, we employed single‐cell sequencing to examine U87‐Cas13a‐EGFRvIII cells after 4 h of crRNA2 or crRNA5 transfection. Cells treated with Lipofectamine 3000 alone were used as controls. The workflow of the scRNA‐seq is shown in Figure S6 (Supporting Information), and the quality control report is shown in Table S2 (Supporting Information). The estimated number of cells in the crRNA2 group was less than half of that in the control and crRNA5 groups, indicating loss of the 3′‐UTR and failure of library building in a large portion of the cells in this group. The median number of genes per cell in the crRNA2 group was 3012, which was ≈75% of that in the other two groups (Figure 2e).

The scRNA‐seq data were further analyzed by tSNE projection. Each cell of the 15 507 cells was grouped into one of the 12 clusters. Cells in the EGFRvIII and crRNA5 groups diffusely distributed into clusters 1, 2, 3, 4, 5, 6, 7, 8, and 10, while most crRNA2 cells were grouped into clusters 9, 11, and 12 (Figure 2f). In total, 430 genes were highly expressed in clusters 9, 11, and 12. The upregulated genes in the crRNA2 group (cluster A: clusters 9, 11, and 12 (Figure S7a,b, Table S3, Supporting Information)) were enriched in oncogenic pathways (including the MAPK and NF‐κB signaling pathways (Figure S7c, Supporting Information)) and malignant biological processes (including apoptosis and immune response‐related terms (Figure S7d, Table S4, Supporting Information)).

To extend the application of the CRISPR‐Cas13a system, we transfected U87‐Cas13a‐EGFRvIII cells with crRNA2, crRNA5, or the Lipofectamine 3000 transfection reagent alone. After 4 h, the cells in each group were collected and injected into mouse brains to establish an intracranial GBM model (**Figure** **3**a). All six mice in the EGFRvIII‐control and crRNA5 groups formed tumors on day 14, while tumors were detected in only four of the six mice in the crRNA2 group. The average luminescence value in the crRNA2 group was only ≈1% of that in the control and crRNA5 groups (Figure 3b). Upon examination on days 21 and 27, the tumors in the control and crRNA5 groups grew steadily, while the tumors in the crRNA2 group barely grew (Figure 3b,c). The immunofluorescence analysis further revealed tumor cell proliferation and angiogenesis in the control and crRNA5 groups but not in the crRNA2 group, confirming the tumor‐inhibiting potential of the CRISPR‐Cas13a system (Figure 3d).

**Figure 3:**
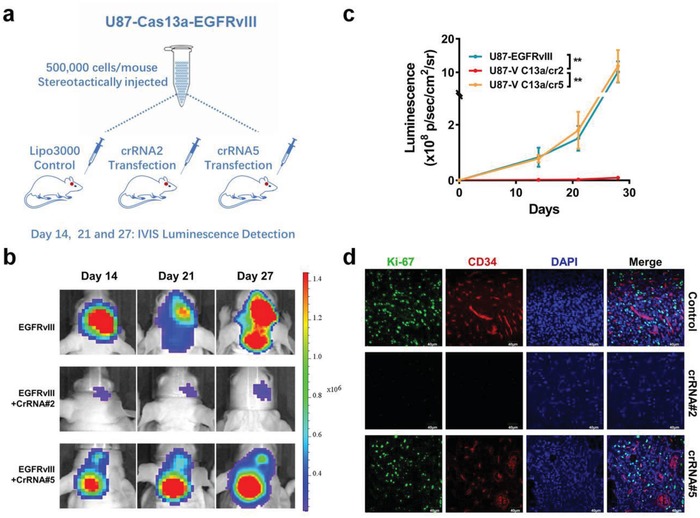
CRISPR‐Cas13a system inhibits the formation of intracranial tumors. a) U87‐Cas13a‐EGFRvIII cells pretreated with crRNA2, crRNA5, or Lipofectamine 3000 (control) for 4 h were implanted in the brains of nude mice. b,c) Tumor formation was assessed by bioluminescence imaging. Bioluminescent signals were measured at days 14, 21, and 27 after implantation. d) The growth of tumors was visualized by immunofluorescence staining for Ki‐67 and CD34.

In summary, we demonstrated that the CRISPR‐Cas13a system induces the collateral effect in human cancer cells. This is the first time that the collateral effect has been reported in eukaryotes.

Abudayyeh et al. reported the collateral effect in *E. coli* but not in human cells.[qv: 2a,10] The differences between their research and ours are as follows: (1) We used lentivirus to express the Cas13a gene in human GBM cancer cells, while they employed a plasmid vector; (2) Cas13a was fused with GFP in their work; (3) crRNA was also expressed by a vector in their research, while we used in vivo transcribed crRNAs and transfected them into cells by Lipofectamine 3000; (4) the target genes in their work were all endogenous genes, whereas we overexpressed GFP and EGFRvIII using a lentivirus.

Jing et al. implemented the CRISPR‐Cas13a system in yeast,^11^ and they used plasmids to overexpress Cas13a. It is interesting to note that the growth of yeast is significantly inhibited when the tdh1 target gene is knocked down. This inhibitory phenomenon is much weaker in the ade6 group, in which the knockdown effect was weak. The authors considered this result to indicate the important cellular function of tdh1. At the same time, Zhao et al. used CRISPR‐Cas13 treatment for KRAS in pancreatic cancer^12^ by cotransfecting Lw.Cas13a protein and crRNAs into cells, but they did not report a collateral effect in their study. CRISPR‐Cas13a can also be used to engineer interference against RNA viruses in plants.^13^ The collateral effect should be due to a RNase that is triggered by some conditions. The extended complementarity between guide and target RNA may block the self‐targeting of Cas13.^14^

However, we are unsure of the specific reason for the different results between our study and others. We analyzed the RNA integrity of the LN229 glioma cell line and HEK293T cells after treatment with the crRNA‐CRISPR/Cas13a system. The Agilent Bioanalyzer 2100 indicated that the collateral effect tended to occur in glioma cells but not in HEK293T cells (Figure S8, Supporting Information). Further research needs to be done to find an explanation. In our study, we exploited the collateral effect of the CRISPR‐Cas13a system to kill GBM cells overexpressing EGFRvIII. Inhibition was also observed in a tumor xenograft model.

Further research is also necessary to clarify the cleavage pattern and mechanism of CRISPR‐Cas13a in glioma cancer cells. Nevertheless, we believe that the CRISPR‐Cas13a system will be of great use in cancer therapy due to its biological characteristics.

## Experimental Section

*Cell Culture and Lentivirus Infection*: Human U87 GBM cells were obtained from American Type Culture Collection and cultured in complete Dulbecco's modified Eagle's medium containing 10% fetal bovine serum, 100 units mL^−1^ penicillin, and 50 µg mL^−1^ streptomycin. Lentivirus containing LwCas13a, nuclease‐dead‐LwCas13a,^2^ EGFRvIII, and EGFP was purchased from GENECHEM (Shanghai, China) and patented in China (201810465791.3). The vector maps are shown in Figure S9 (Supporting Information). The lentivirus was added according to the multiplicity of infection (MOI) of U87 cells (MOI = 5). The positive Cas13a‐transfected cells were selected by puromycin for one week and then transfected the cells with either EGFP or EGFRvIII for at least 48 h following the manual. The concentration of puromycin was 4 µg mL^−1^ for selection and 2 µg mL^−1^ for maintenance. The transfected cells were used for less than one month in case the target gene was lost.

*crRNA Design, In Vitro Transcription, and crRNA Transfection*: The crRNAs used were synthesized in vitro by Integrated Biotech Solutions, Shanghai, China. crRNAs were designed using the CRISPR‐RT Design Tool (http://bioinfolab.miamioh.edu/CRISPR-RT) with the following parameters: PFS sequence on target A, U, or C; PFS sequence off target A, U, or C; length of the target complementary region of crRNA was 28 nt; length of the seed region was 10 nt; and an off‐target basic setting was chosen.

The following nontargeting guide sequences were obtained from previously published reports:^3^ nontargeting spacer 1, CAGACTATGCGTCGACAAGCCAGGCATT; and nontargeting spacer 2, CTGGTCAGACTTTATAACGGCCAAGGTT. The following sequences were used to generate nucleic acid targets:

EGFP crRNA, AAGCACUGCACGCCGUAGGUCAGGGUGG;

EGFRvIII,

crRNA1, CCACAUAAUUACCUUUCUUUUCCUCCAG;

crRNA2, CACCACAUAAUUACCUUUCUUUUCCUCC;

crRNA3, UCACCACAUAAUUACCUUUCUUUUCCUC;

crRNA4, GAUCUGUCACCACAUAAUUACCUUUCUU;

crRNA5, UGAUCUGUCACCACAUAAUUACCUUUCU.

To generate nucleic acid targets, oligonucleotides were amplified by PCR. dsDNA amplicons were extracted from a gel and purified using a MinElute gel extraction kit (Qiagen). The resulting purified dsDNA was transcribed via overnight incubation at 30 °C with a HiScribe T7 Quick High Yield RNA Synthesis kit (New England Biolabs). Transcribed RNA was purified using a MEGAclear Transcription Clean‐up kit (Thermo Fisher). All RNA targets used in this study are listed in the Supporting Information. To generate crRNAs, oligonucleotides were ordered as DNA (IBSBIO) with an additional 5′ T7 promoter sequence. crRNA template DNA was annealed with a T7 primer and transcribed via overnight incubation at 37 °C with a HiScribe T7 Quick High Yield RNA Synthesis kit (New England Biolabs), and 150 ng mL^−1^ crRNA was then transfected with Lipofectamine R3000 (Invitrogen, California, USA) following the manufacturer's instructions.

*Immunofluorescence Analysis, Western Blotting, and ELISA*: U87 cells were transduced with the indicated lentivirus and crRNAs. Cells were subjected to immunofluorescence and Western blot analyses, while the serum was used for ELISA. Nonspecific binding was blocked using 10% goat serum. Primary antibodies against β‐tubulin, OAS1, and RNase L were purchased from Proteintech (Illinois, USA; dilution 1:1000). Primary antibodies against Ki‐67 (Zsbio, Beijing, China; dilution 1:100) and CD34 (Abcam, Cambridge, UK; dilution 1:200) were used. Phalloidin was purchased from CST (Danvers, USA; dilution 1:200). The indicated primary antibody was incubated with cells overnight at 4 °C followed by room‐temperature incubation with an appropriate secondary antibody (Alexa Fluor antibody (Invitrogen, USA; 1:200) and GAPDH (Abcam 1:2000). OAS1 ELISA and TNF‐α ELISA kits were purchased from Lanpaibio (Shanghai, China), and they were performed according to the manufacturer's protocol.

*qRT‐PCR*: Treated cells were lysed in TRIzol reagent (Invitrogen, California, USA). The lysate was well mixed with chloroform and centrifuged at 13 000 rpm for 10 min at 4 °C. The upper, aqueous phase was transferred to a clean tube, and an equal volume of isopropanol was added. The samples were mixed well by shaking and stored at −20 °C overnight. Total RNA was precipitated and used for qRT‐PCR. cDNA was synthesized from 2 µg of total RNA using a reverse transcription kit purchased from Promega (Madison, WI) following the manufacturer's protocol. qRT‐PCR was performed using SYBR Green Master Mix from Life Technologies (Carlsbad, CA). Amplification was performed with the DNA Engine Opticon 2 Two‐Color qRT‐PCR detection system (Bio‐Rad Laboratories, Hercules, CA).

The following primers were used:

EGFP‐F, CCCGACAACCACTACCTGAG;

EGFP‐R, GTCCATGCCGAGAGTGATCC;

EGFRvIII‐F, GGCTCTGGAGGAAAAGAAAGGTAAT;

EGFRvIII‐R, TCCTCCATCTCATAGCTGTCG;

h‐GAPDH‐F, TGCACCACCAACTGCTTAGC;

h‐GAPDH‐R, GGCATGGACTGTGGTCATGAG;

m‐GAPDH‐F, CATGGCCTTCCGTGTTCCT;

m‐GAPDH‐R, CCTGCTTCACCACCTTCTTGA;

HOTAIR‐F, ATAGGCAAATGTCAGAGGGTT;

HOTAIR‐R, TCTTAAATTGGGCTGGGTC;

L3MBTL1‐F, AGAGGGACAACCCACTGCTA;

L3MBTL1‐R, GGCCTTCTGCTCCTCTAGGT;

Cas13a‐F, TGGAAAAGTACCAGTCCGCC; and

Cas13a‐R, TCGAAGTCCTCGGTCACTCT.

*RNA‐Denaturing Gel Electrophoresis*: Total RNA was prepared as described above. A 1% agarose gel was prepared with 2.4 g of agarose, 24 mL of 10× MOPS buffer, 12.6 mL of 37–40% formalin, and 204 mL of distilled water. After polymerizing, the gel was prerun in 1× MOPS buffer for 10 min at 80 V, and 5 µg of total RNA was then separated. Electrophoresis was stopped when the blue dye front reached half the length of the gel. The G:BOX F3 gel imaging system was employed to analyze the RNA bands.

*RNA‐Seq Analysis*: U87‐Cas13a‐EGFRvIII cells were transfected with crRNA2 and lysed in TRIzol reagent at 0, 2, 4, and 8 h. Three triplicate samples were collected, and each sample was sequenced twice. Total RNA was sequenced using a BGISEQ‐500. PCA was performed to assess expression patterns of grouped samples with R programming language (http://cran.r-project.org). Differentially expressed genes were screened using the DESeq2 algorithm. Genes with adjusted *P* < 0.05 and fold change >2 were regarded as candidate differential genes, and they were subjected to subsequent enrichment analysis. Gene Ontology visualization was performed with the BiNGO plugin embedded in Cytoscape software.

*Single‐Cell RNA‐Seq*: U87‐Cas13a‐EGFRvIII cells were transfected with crRNA2, crRNA5, or Lipofectamine 3000 for 4 h. Cells were trypsinized and resuspended in phosphate buffered saline (PBS) containing 0.04% weight/volume bovine serum albumin. Barcoded signal‐cell gel beads‐in‐emulsion (GEMs) were created by 10× Genomics Chromium. GEMs were then reverse‐transcribed to generate single‐cell RNA‐seq libraries. cDNAs in the scRNA‐seq libraries were then amplified and sequenced. Analyses of single‐cell transcriptomes were conducted using the LOUPE cell browser.

*Real‐Time Cell Proliferation Assay*: A total of 2000 cells (100 µL/well) were seeded into an E‐Plate 16. After 20 h of incubation, cells were transfected with crRNA 1–5 as indicated. Cell proliferation was automatically monitored in each well using the xCELLigence system. The cell index (CI) was recorded as an indicator of cell proliferation. RTCA software (version 2.0) was employed to automatically calculate the CI values every 15 min up to 100 h. Data were processed using RTCA software.

*Nude Mouse Glioma Intracranial Model*: U87‐Cas13a‐EGFRvIII cells were transfected with 150 ng mL^−1^ crRNA2, crRNA5, or Lipofectamine 3000 for 4 h. Eighteen 4‐week‐old BALB/c‐nu female mice were divided randomly into the following three groups: control, crRNA2, and crRNA5. Then, 500 000 cells were stereotactically injected into the right corpus striatum of nude mice. Intracranial tumor growth was monitored using a bioluminescence imaging system on days 14, 21, and 27. Mouse whole brains were harvested on day 28 and cut into 8 µm thick frozen coronal sections. All animal experiments were reviewed and approved by the Animal Ethical and Welfare Committee (AEWC), Tianjin Medical University (accreditation number: TMUaMEC 2018034).

## Conflict of Interest

The authors declare no conflict of interest.

## Supporting information

Supplementary

Supplementary

Supplementary

Supplementary

Supplementary
