# Supplementary material for: The CRISPR‐Cas13a Gene‐Editing System Induces Collateral Cleavage of RNA in Glioma Cells
Source: Adv Sci (Weinh). 2019 Aug 29;6(20):1901299. doi: 10.1002/advs.201901299 (PMC6794629; doi:10.1002/advs.201901299)
Supplement: Supplementary file 1 — Supplementary [file ADVS-6-1901299-s001.pdf]

Copyright WILEY-VCH Verlag GmbH & Co. KGaA, 69469 Weinheim, Germany, 2019.

ADVANCED SCIENCE
Open Access

Supporting Information

for *Adv. Sci.,* DOI: 10.1002/advs.201901299

The CRISPR-Cas13a Gene-Editing System Induces Collateral Cleavage of RNA in Glioma Cells

*Qixue Wang, Xing Liu, Junhu Zhou, Chao Yang, Guangxiu Wang, Yanli Tan, Ye Wu, Sijing Zhang, Kaikai Yi, and Chunsheng Kang**

Supporting Information

The CRISPR-Cas13a gene-editing system induces collateral cleavage of RNA in human cancer cells

*Qixue Wang, Xing Liu, Junhu Zhou, Chao Yang, Guangxiu Wang, Yanli Tan, Ye Wu, Sijing Zhang, Kaikai Yi, Chunsheng Kang**

a

EGFP

	Ct Value		
0 h	23.68	23.17	23.43
2 h	25.28	24.75	25.80
4 h	25.37	26.04	26.71
8 h	25.65	25.28	26.02

GAPDH

	Ct Value		
0 h	13.69	13.37	13.32
2 h	13.79	14.07	13.54
4 h	14.93	14.91	14.78
8 h	15.34	15.15	15.28

b

L3MBTL1

	Ct Value		
0 h	26.83	26.70	26.77
2 h	27.74	27.37	27.26
4 h	27.91	28.12	27.92
8 h	28.36	28.22	28.26

HOTAIR

	Ct Value		
0 h	25.80	25.95	26.15
2 h	25.29	25.20	25.27
4 h	26.20	26.24	26.01
8 h	26.35	26.23	26.38

Supplement Figure 1. Ct values from qRT-PCR of EGFP, GAPDH (a), HOTAIR and L3MBTL1 (b) at the indicated times.

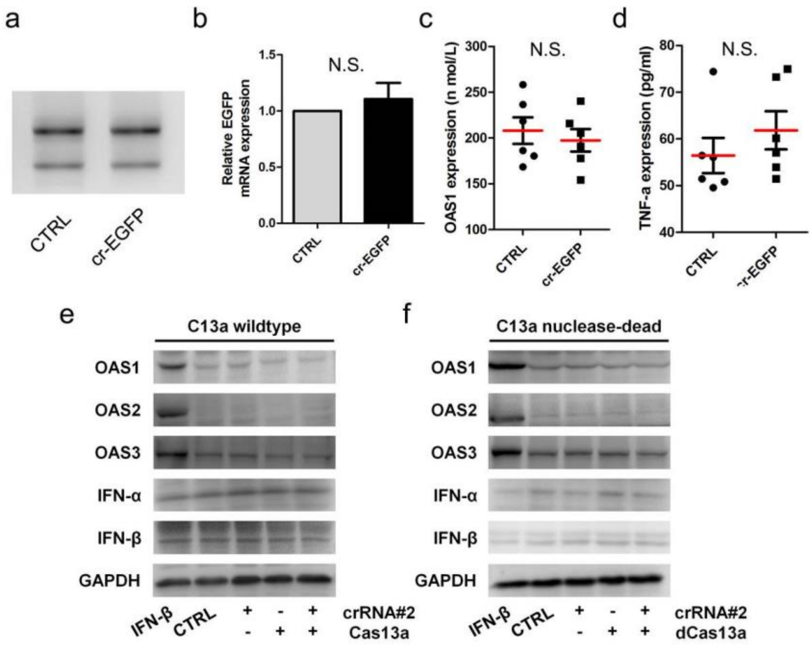

Supplement Figure 2. The CRISPR/Cas13a system does not trigger an innate immune response. (a) RNA-denaturing gel electrophoresis, (b) qRT-PCR and (c) ELISA were employed to examine RNA changes as well as OAS1 and TNF-α in the culture medium of U87 cells transfected with crRNAs but without Cas13a. (d, e) Western blot analysis was used to examine OAS1~3 and IFN-α, β in U87 cells after the indicated treatment. The lentiviral transduction time for OAS and IFN Western blot measurement was 48 h following 8 h of crRNA transfection.

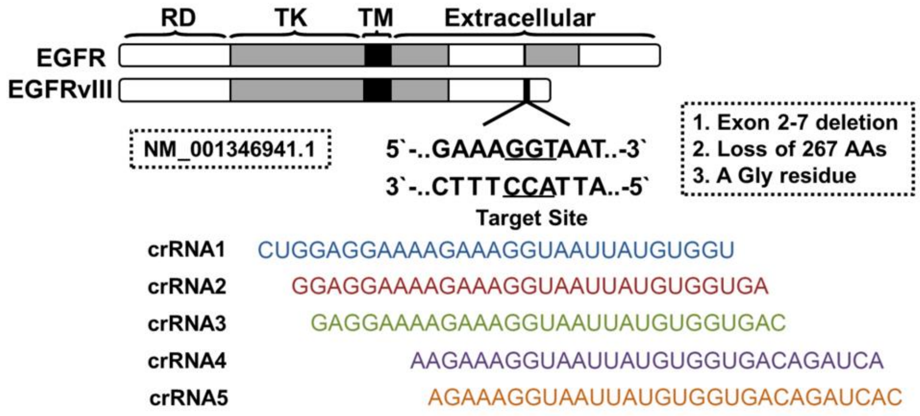

Supplement Figure 3. Scheme of the crRNA design for EGFRvIII.

a

	qPCR Cycle Threshold			
	4h-vIII	8h-vIII	4h-GAPDH	8h-GAPDH
Control	22.30000	22.05667	14.84333	14.56667
crRNA#1	22.03667	22.46333	14.73667	14.05333
crRNA#2	23.45333	24.94000	16.31667	19.35333
crRNA#3	22.40333	22.86000	15.05000	15.46333
crRNA#4	22.23667	22.06667	14.82667	14.64333
crRNA#5	22.37000	23.35333	14.72333	14.97333

b

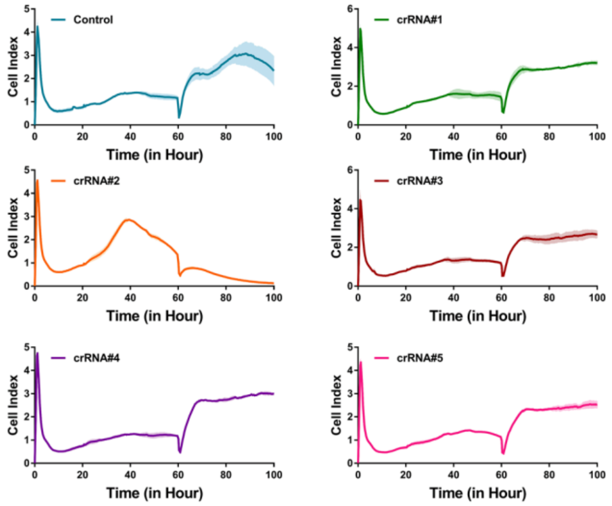

Supplement Figure 4. (a) qRT-PCR Ct values of U87-Cas13a-EGFRvIII after crRNA 1-5 treatment at the indicated time. (b) Cell proliferation was determined by RTCA. The recording time was 100 h.

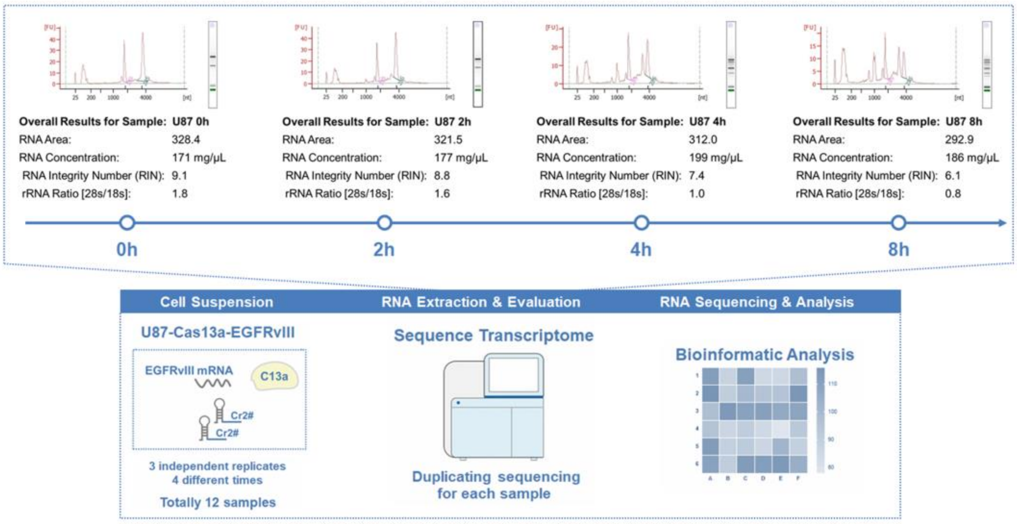

Supplement Figure 5. RNA-seq quality control.

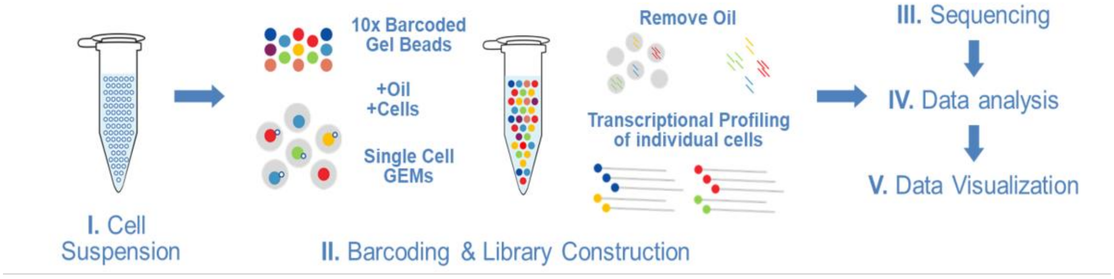

Supplement Figure 6. Schematic illustration of single-cell RNA-seq.

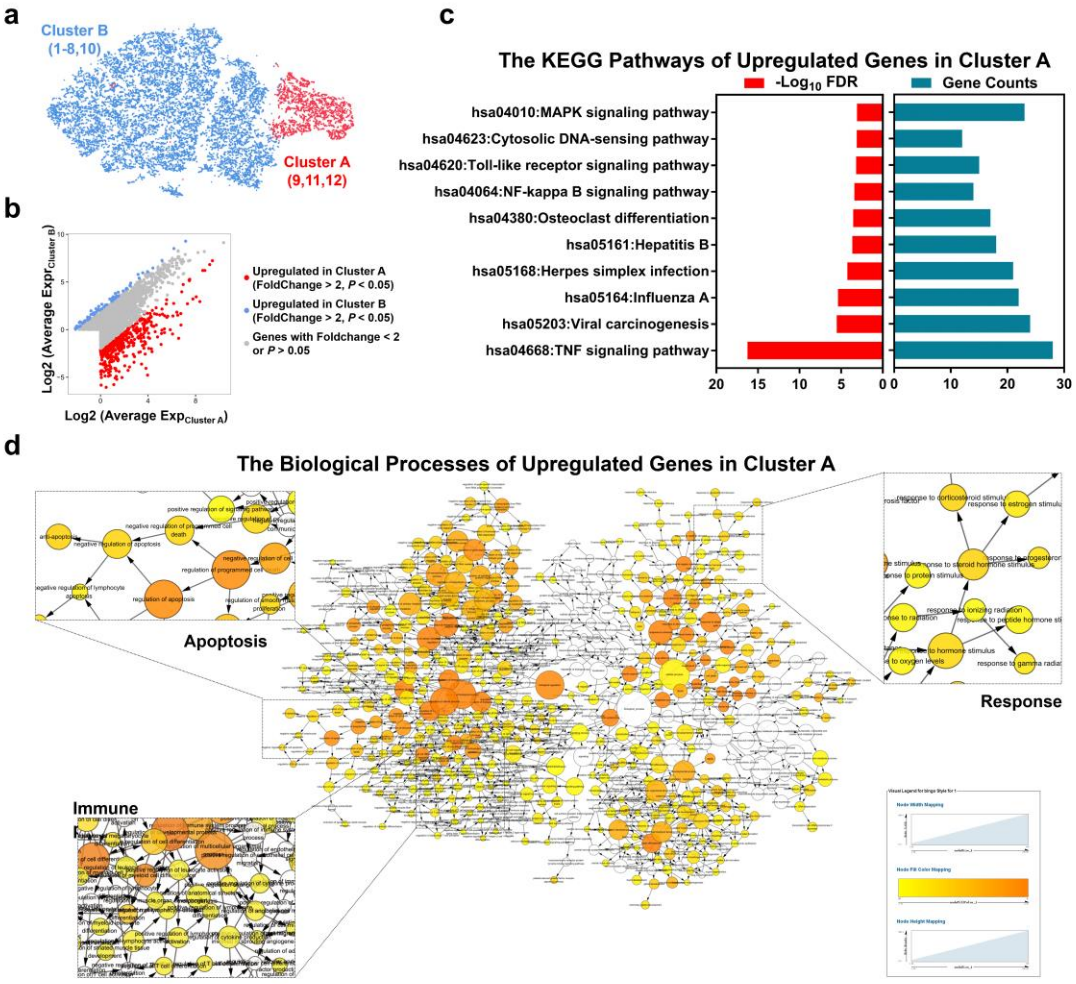

Supplement Figure 7. Identification and gene annotation of differentially expressed genes in crRNA 2-treated U87 cells. (a) A cluster of U87 cells was specifically distributed in the crRNA2 group. (b) The differentially expressed genes between cluster A and cluster B. (c) KEGG pathway of cluster A genes. (d) Biological processes of genes in cluster A.

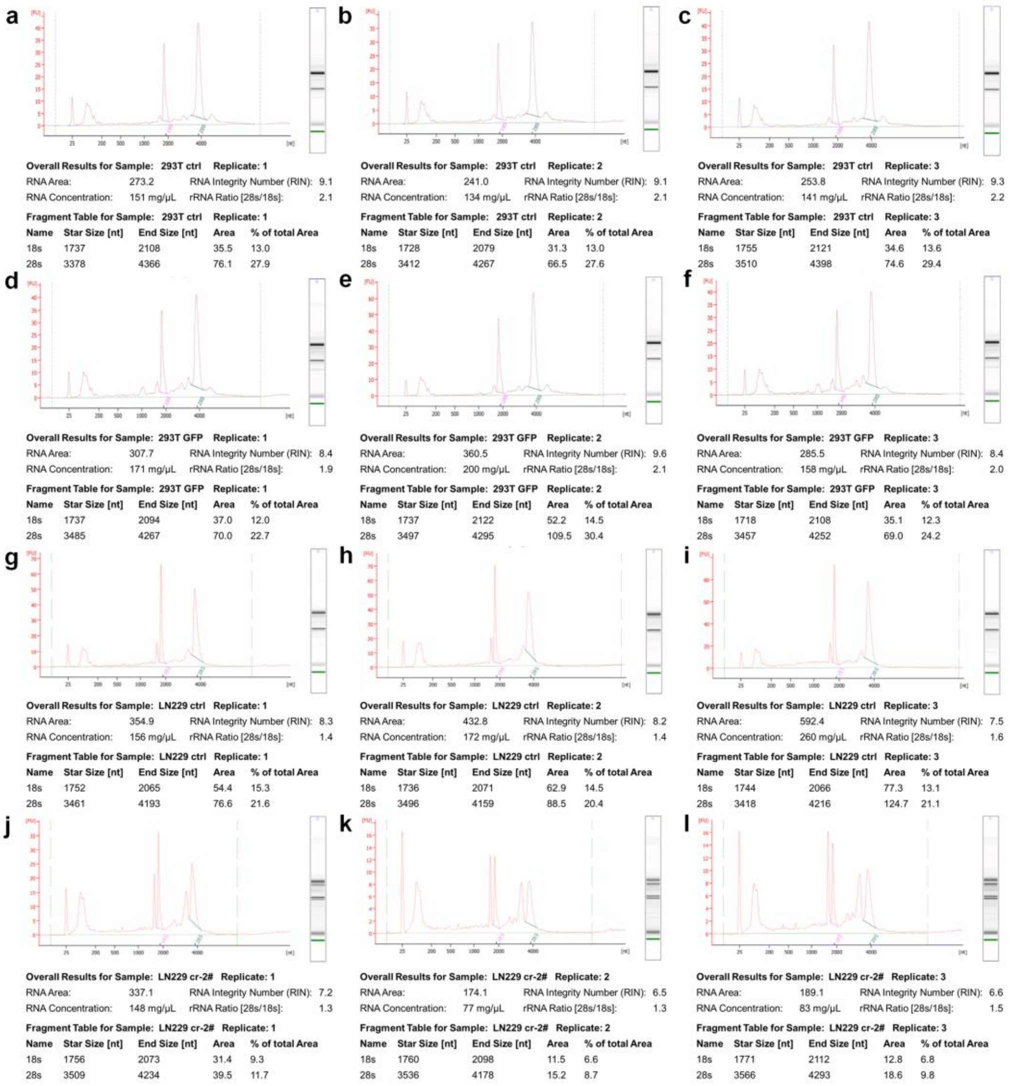

Supplement Figure 8. The CRISPR/Cas13a system identifies a collateral effect in glioma cells. RNA quality control of 293T-EGFP cells from the control group (a, b, c) and the cr-EGFP treatment group (d, e, f). LN229-EGFRvIII cells from the control group (g, h, i) and the cr-2# treatment group (j, k, l). RNA quality was determined by an Agilent Bioanalyzer 2100.

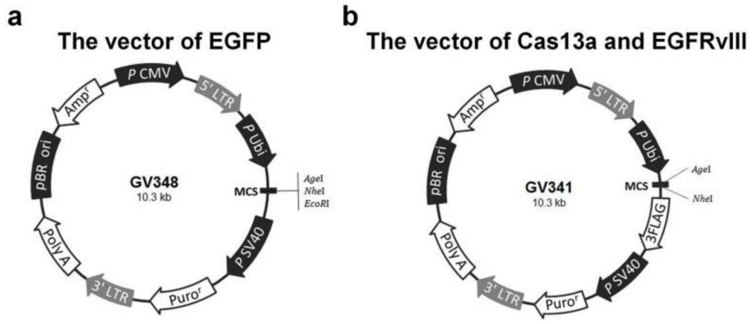

Supplement Figure 9. Lentiviral vector maps.

Supplement Table 1. Differential genes of U87 cells receiving CRISPR-Cas13a treatment at 2, 4 and 8 h according to RNA sequencing.

Supplement Table 2. Quality control report of single-cell RNA sequencing.

Supplement Table 3. Differentially expressed genes between cluster A and cluster B in the single-cell RNA sequencing data.

Supplement Table 4 Biological processes of upregulated genes in cluster A.
